# Bis(1-methyl-1*H*-imidazole-κ*N* ^3^)[*N*,*N*′-*o*-phenyl­enebis(pyridine-2-carbox­amido)-κ^4^ *N*]manganese(II)

**DOI:** 10.1107/S1600536808041640

**Published:** 2008-12-17

**Authors:** Zaki N. Zahran, Nan Xu, Douglas R. Powell, George B. Richter-Addo

**Affiliations:** a Chemistry Department, Faculty of Science, Tanta University, Tanta, Egypt; b Department of Chemistry and Biochemistry, University of Oklahoma, 620 Parrington Oval, Norman, OK 73019 USA

## Abstract

The title compound, [Mn(C_18_H_12_N_4_O_2_)(C_4_H_6_N_2_)_2_], belongs to the family of 1,2-bis­(pyridine-2-carboxamido)benzene (H_2_bpb) ligated metal complexes. The manganese center is octa­hedrally coordinated by a bpb ligand and two axial 1-methyl­imidazole mol­ecules. The axial N—Mn—N group is bent with a bond angle of 151.79 (7)°.

## Related literature

For the structures of related Mn complexes, see Liang *et al.* (2007 ▶), Lin *et al.* (2003 ▶), and Havranek *et al.* (1999 ▶).
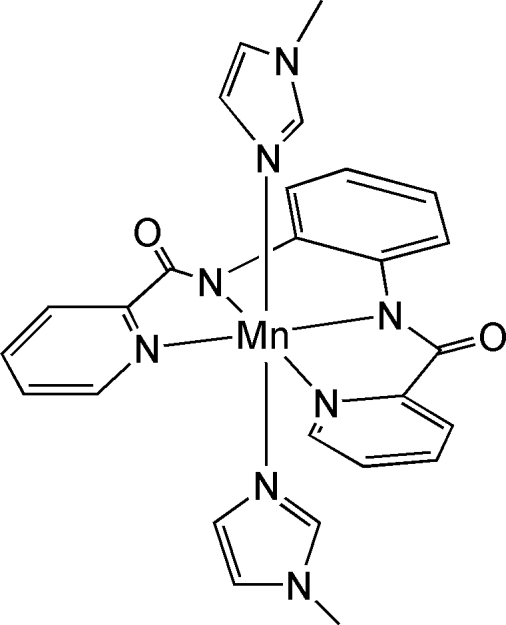


## Experimental

### 

#### Crystal data

[Mn(C_18_H_12_N_4_O_2_)(C_4_H_6_N_2_)_2_]
*M* *_r_* = 535.47
Orthorhombic, 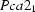

*a* = 13.819 (3) Å
*b* = 9.894 (2) Å
*c* = 17.864 (4) Å
*V* = 2442.5 (9) Å^3^
*Z* = 4
Mo *K*α radiation
μ = 0.58 mm^−1^
*T* = 100 (2) K
0.54 × 0.35 × 0.04 mm

#### Data collection

Bruker APEX diffractometer
Absorption correction: multi-scan (*SADABS*; Sheldrick, 2007 ▶) *T*_min_ = 0.744, *T*_max_ = 0.977
8288 measured reflections
3473 independent reflections
3301 reflections with *I* > 2σ(*I*)
*R*_int_ = 0.022

#### Refinement

*R*[*F* ^2^ > 2σ(*F* ^2^)] = 0.025
*wR*(*F* ^2^) = 0.062
*S* = 1.02
3473 reflections
335 parameters
1 restraint
H-atom parameters constrained
Δρ_max_ = 0.33 e Å^−3^
Δρ_min_ = −0.19 e Å^−3^
Absolute structure: Flack (1983 ▶), 989 Friedel pairs
Flack parameter: 0.046 (17)

### 

Data collection: *SMART* (Bruker, 1998 ▶); cell refinement: *SAINT* (Bruker, 1998 ▶); data reduction: *SAINT*; program(s) used to solve structure: *SHELXTL* (Sheldrick, 2008 ▶); program(s) used to refine structure: *SHELXTL*; molecular graphics: *SHELXTL*; software used to prepare material for publication: *SHELXTL*.

## Supplementary Material

Crystal structure: contains datablocks I, global. DOI: 10.1107/S1600536808041640/pk2130sup1.cif

Structure factors: contains datablocks I. DOI: 10.1107/S1600536808041640/pk2130Isup2.hkl

Additional supplementary materials:  crystallographic information; 3D view; checkCIF report

## Figures and Tables

**Fig. 1.**
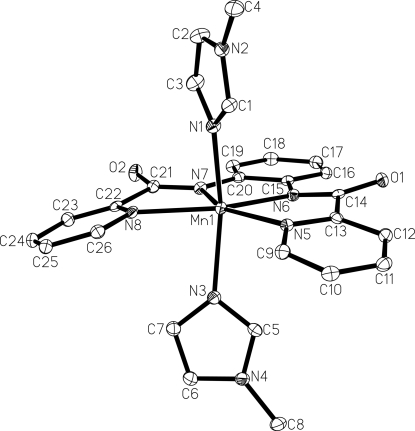
The molecular structure showing the atom-numbering scheme. Displacement ellipsoids are drawn at the 35% probability level (H atoms omitted for clarity).

## supplementary crystallographic information

### Comment

In this paper, we report the structure of the title compound, a six-coordinate {bis(1-methylimidazole)}(bpb)manganese(II) (H_2_bpb = 1,2-bis(pyridine-2-carboxamido)benzene). To the best of our knowledge, this is the first reported structure of a Mn(II) complex containing ligated bpb or its derivatives. The structures of the related [Mn(bpb)(H_2_O)Cl] (Lin *et al.* 2003), [Mn(bpc)(DMF)Cl] (H_2_bpc = 1,2-bis(pyridine-2-carboxamido)-4,5-dichlorobenzene) (Liang *et al.*,2007) and [Mn(bpmb)(OMe)(OCOCH_3_)] (H_2_bpmb = *N,N'*-bis(pyridine-2-ylcarbonyl)-4-methoxycarbonylbenzene-1,2-diamine) (Havranek *et al.*, 1999) complexes have been reported previously.

The molecular structure is shown in Fig. 1. The manganese center is six-coordinate, displaying a distorted octahedral geometry. A bpb ligand binds to the manganese through its two deprotonated amide N atoms and two pyridyl N atoms. The two axial positions are occupied by 1-methylimidazole molecules. The Mn—N(pyridyl) distances of 2.2621 (19) Å and 2.2684 (19)Å are longer than the Mn—*N*(amide) distances at 2.1764 (19) Å and 2.1794 (18) Å. Both of the Mn—N(pyridyl) and Mn—*N*(amide) distances are significantly longer than those in the related Mn complexes reported previously (Liang *et al.*, 2007; Lin *et al.*, 2003; Havranek *et al.*, 1999). In addition, the C13—C14 and C21—C22 distances of 1.513 (3) Å and 1.526 (3) Å are slightly longer than those of the other Mn complexes mentioned above. Mn—*N*(1-methylimidazole) distances are 2.2552 (19) Å and 2.280 (2) Å. The axial N—Mn—N linkage is bent with a bond angle of 151.79 (7)°.

### Experimental

To a CH_2_Cl_2_ suspension (20 ml) of Mn(bpb)Cl (0.2 g, 0.49 mmol) was added excess piperidine (2 ml, 0.02 mol) (Aldrich Chemical Company, used as received) and then purged with nitric oxide (98%; Matheson Gas, purified by passing through KOH pellets and a cold trap (dry ice/acetone)) for 30 min. This resulted in the precipitation of a red-brown intermediate Mn(bpb)(NO)(pip) (ν_NO_ 1732 cm^-1^; KBr pellet) that was isolated by filtration. This intermediate was redissolved in CH_2_Cl_2_, and excess 1-methylimidazole (0.2 ml, 2.6 mmol) (Aldrich Chemical Company, used as received) was added. The resulting mixture was stirred for 30 min. A brown solid was obtained after removal of the solvent under vacuum. A suitable red plate-shaped crystal was grown by slow evaporation of a CH_2_Cl_2_ solution of the complex in the presence of excess 1-methylimidazole at room temperature under N_2_.

### Refinement

H atoms were positioned geometrically and refined using a riding model with C—H = 0.95 Å for aromatic carbons, 0.98 Å for methyl carbons. U_iso_(H) values were set to either 1.5U_eq_ (RCH_3_) or 1.2U_eq_ of the attached atom.

### Crystal data

**Table:**

[Mn(C_18_H_12_N_4_O_2_)(C_4_H_6_N_2_)_2_]	*F*(000) = 1108
*M**_r_* = 535.47	*D*_x_ = 1.456 Mg m^−^^3^
Orthorhombic, *P**c**a*2_1_	Mo *K*α radiation, λ = 0.71073 Å
Hall symbol: P 2c -2ac	Cell parameters from 6910 reflections
*a* = 13.819 (3) Å	θ = 2.5–28.3°
*b* = 9.894 (2) Å	µ = 0.58 mm^−^^1^
*c* = 17.864 (4) Å	*T* = 100 K
*V* = 2442.5 (9) Å^3^	Plate, red
*Z* = 4	0.54 × 0.35 × 0.04 mm

### Data collection

**Table:**

Bruker APEX diffractometer	3473 independent reflections
Radiation source: fine-focus sealed tube	3301 reflections with *I* > 2σ(*I*)
graphite	*R*_int_ = 0.022
ω scans	θ_max_ = 26.0°, θ_min_ = 2.1°
Absorption correction: multi-scan (*SADABS*; Sheldrick, 2007)	*h* = −16→17
*T*_min_ = 0.744, *T*_max_ = 0.977	*k* = −12→7
8288 measured reflections	*l* = −22→13

### Refinement

**Table:**

Refinement on *F*^2^	Secondary atom site location: difference Fourier map
Least-squares matrix: full	Hydrogen site location: inferred from neighbouring sites
*R*[*F*^2^ > 2σ(*F*^2^)] = 0.025	H-atom parameters constrained
*wR*(*F*^2^) = 0.062	*w* = 1/[σ^2^(*F*_o_^2^) + (0.038*P*)^2^ + 0.1*P*] where *P* = (*F*_o_^2^ + 2*F*_c_^2^)/3
*S* = 1.02	(Δ/σ)_max_ < 0.001
3473 reflections	Δρ_max_ = 0.33 e Å^−^^3^
335 parameters	Δρ_min_ = −0.19 e Å^−^^3^
1 restraint	Absolute structure: Flack (1983), 989 Friedel pairs
Primary atom site location: structure-invariant direct methods	Flack parameter: 0.046 (17)

### Special details

**Table:**

Geometry. All e.s.d.'s (except the e.s.d. in the dihedral angle between two l.s. planes) are estimated using the full covariance matrix. The cell e.s.d.'s are taken into account individually in the estimation of e.s.d.'s in distances, angles and torsion angles; correlations between e.s.d.'s in cell parameters are only used when they are defined by crystal symmetry. An approximate (isotropic) treatment of cell e.s.d.'s is used for estimating e.s.d.'s involving l.s. planes.

### Fractional atomic coordinates and isotropic or equivalent isotropic displacement parameters (Å^2^)

**Table:**

	*x*	*y*	*z*	*U*_iso_*/*U*_eq_	
Mn1	0.38539 (2)	0.37818 (3)	0.51871 (2)	0.01386 (8)	
O1	0.36109 (12)	0.12118 (15)	0.32716 (9)	0.0186 (3)	
O2	0.65501 (11)	0.32649 (17)	0.63464 (9)	0.0236 (4)	
N1	0.30418 (13)	0.22376 (19)	0.58708 (11)	0.0194 (4)	
N2	0.19272 (14)	0.08610 (19)	0.63164 (11)	0.0201 (4)	
N3	0.40259 (14)	0.58964 (19)	0.46979 (11)	0.0185 (4)	
N4	0.41706 (15)	0.74233 (19)	0.38026 (12)	0.0204 (4)	
N5	0.25565 (14)	0.37585 (17)	0.44149 (10)	0.0157 (4)	
N6	0.42608 (13)	0.24628 (19)	0.42643 (11)	0.0151 (4)	
N7	0.53284 (12)	0.31457 (18)	0.54386 (10)	0.0156 (4)	
N8	0.42419 (13)	0.48437 (18)	0.62717 (10)	0.0156 (4)	
C1	0.21158 (16)	0.1926 (2)	0.58814 (13)	0.0206 (5)	
H1	0.1635	0.2405	0.5610	0.025*	
C2	0.27962 (17)	0.0447 (3)	0.66076 (15)	0.0283 (6)	
H2	0.2902	−0.0294	0.6935	0.034*	
C3	0.34717 (18)	0.1303 (2)	0.63362 (15)	0.0260 (5)	
H3	0.4143	0.1267	0.6449	0.031*	
C4	0.09803 (17)	0.0263 (3)	0.64689 (15)	0.0266 (5)	
H4A	0.0520	0.0549	0.6083	0.040*	
H4B	0.0751	0.0564	0.6961	0.040*	
H4C	0.1035	−0.0724	0.6465	0.040*	
C5	0.38396 (16)	0.6197 (2)	0.39912 (15)	0.0183 (5)	
H5	0.3509	0.5611	0.3656	0.022*	
C6	0.45966 (18)	0.7961 (2)	0.44268 (14)	0.0239 (5)	
H6	0.4896	0.8822	0.4470	0.029*	
C7	0.45043 (16)	0.7012 (2)	0.49738 (13)	0.0216 (5)	
H7	0.4735	0.7105	0.5472	0.026*	
C8	0.4078 (2)	0.8059 (2)	0.30677 (15)	0.0271 (5)	
H8A	0.3726	0.7453	0.2730	0.041*	
H8B	0.4724	0.8239	0.2863	0.041*	
H8C	0.3723	0.8912	0.3117	0.041*	
C9	0.17954 (16)	0.4603 (2)	0.44273 (13)	0.0183 (5)	
H9	0.1700	0.5150	0.4858	0.022*	
C10	0.11453 (15)	0.4712 (2)	0.38434 (14)	0.0200 (5)	
H10	0.0623	0.5334	0.3865	0.024*	
C11	0.12757 (17)	0.3891 (2)	0.32266 (15)	0.0206 (5)	
H11	0.0835	0.3932	0.2819	0.025*	
C12	0.20533 (16)	0.3006 (2)	0.32047 (13)	0.0188 (5)	
H12	0.2149	0.2430	0.2785	0.023*	
C13	0.26922 (15)	0.2974 (2)	0.38068 (13)	0.0158 (4)	
C14	0.35901 (16)	0.2101 (2)	0.37724 (12)	0.0147 (4)	
C15	0.51905 (15)	0.1872 (2)	0.43010 (13)	0.0151 (4)	
C16	0.55961 (17)	0.1028 (2)	0.37494 (14)	0.0178 (5)	
H16	0.5224	0.0787	0.3323	0.021*	
C17	0.65348 (17)	0.0543 (2)	0.38227 (14)	0.0212 (5)	
H17	0.6800	−0.0026	0.3447	0.025*	
C18	0.70854 (17)	0.0888 (2)	0.44413 (14)	0.0216 (5)	
H18	0.7723	0.0542	0.4492	0.026*	
C19	0.67119 (15)	0.1736 (2)	0.49883 (13)	0.0193 (5)	
H19	0.7102	0.1978	0.5405	0.023*	
C20	0.57629 (15)	0.2242 (2)	0.49336 (13)	0.0163 (5)	
C21	0.57588 (16)	0.3589 (2)	0.60555 (13)	0.0171 (5)	
C22	0.51797 (15)	0.4679 (2)	0.64610 (12)	0.0160 (4)	
C23	0.56201 (16)	0.5497 (2)	0.69906 (13)	0.0200 (5)	
H23	0.6280	0.5363	0.7119	0.024*	
C24	0.50893 (18)	0.6512 (2)	0.73299 (13)	0.0234 (5)	
H24	0.5385	0.7100	0.7683	0.028*	
C25	0.41228 (18)	0.6657 (2)	0.71479 (14)	0.0213 (5)	
H25	0.3737	0.7328	0.7383	0.026*	
C26	0.37272 (17)	0.5799 (2)	0.66128 (14)	0.0187 (5)	
H26	0.3063	0.5898	0.6486	0.022*	

### Atomic displacement parameters (Å^2^)

**Table:**

	*U*^11^	*U*^22^	*U*^33^	*U*^12^	*U*^13^	*U*^23^
Mn1	0.01291 (14)	0.01638 (15)	0.01230 (15)	−0.00013 (12)	−0.00020 (14)	−0.00077 (16)
O1	0.0198 (8)	0.0179 (8)	0.0181 (9)	−0.0009 (6)	0.0002 (7)	−0.0041 (7)
O2	0.0145 (8)	0.0335 (9)	0.0227 (9)	0.0036 (7)	−0.0046 (7)	−0.0034 (8)
N1	0.0194 (10)	0.0207 (10)	0.0181 (10)	−0.0003 (8)	0.0007 (8)	0.0018 (8)
N2	0.0224 (10)	0.0204 (10)	0.0175 (10)	−0.0027 (8)	0.0029 (8)	−0.0006 (8)
N3	0.0208 (10)	0.0178 (9)	0.0170 (10)	0.0010 (7)	0.0003 (8)	0.0005 (8)
N4	0.0258 (10)	0.0165 (10)	0.0188 (10)	0.0017 (8)	0.0023 (8)	0.0012 (8)
N5	0.0172 (9)	0.0148 (9)	0.0149 (10)	−0.0009 (7)	0.0003 (8)	0.0010 (7)
N6	0.0153 (9)	0.0161 (8)	0.0138 (9)	0.0004 (7)	0.0029 (7)	0.0003 (7)
N7	0.0120 (9)	0.0188 (9)	0.0161 (9)	0.0009 (7)	0.0013 (7)	0.0014 (7)
N8	0.0171 (9)	0.0181 (9)	0.0116 (9)	−0.0019 (7)	0.0007 (7)	0.0020 (8)
C1	0.0217 (12)	0.0219 (12)	0.0181 (12)	−0.0003 (10)	0.0002 (9)	0.0028 (9)
C2	0.0294 (13)	0.0272 (13)	0.0282 (14)	0.0023 (10)	0.0008 (11)	0.0096 (11)
C3	0.0206 (12)	0.0314 (14)	0.0259 (13)	0.0031 (10)	−0.0013 (11)	0.0050 (11)
C4	0.0241 (12)	0.0291 (13)	0.0266 (13)	−0.0073 (10)	0.0035 (10)	0.0032 (11)
C5	0.0177 (12)	0.0151 (12)	0.0220 (13)	−0.0017 (8)	0.0020 (9)	−0.0010 (9)
C6	0.0309 (13)	0.0188 (11)	0.0220 (13)	−0.0047 (10)	0.0013 (10)	0.0000 (10)
C7	0.0231 (11)	0.0213 (12)	0.0203 (12)	−0.0039 (9)	0.0017 (9)	−0.0002 (9)
C8	0.0400 (15)	0.0218 (12)	0.0197 (12)	0.0026 (11)	0.0031 (11)	0.0045 (10)
C9	0.0183 (11)	0.0192 (11)	0.0174 (11)	0.0009 (9)	0.0038 (9)	−0.0002 (9)
C10	0.0158 (11)	0.0207 (11)	0.0235 (12)	0.0008 (9)	0.0015 (9)	0.0040 (10)
C11	0.0188 (12)	0.0225 (12)	0.0206 (12)	−0.0035 (9)	−0.0038 (9)	0.0054 (10)
C12	0.0213 (12)	0.0187 (11)	0.0163 (11)	−0.0027 (9)	0.0005 (9)	−0.0002 (9)
C13	0.0178 (11)	0.0124 (10)	0.0172 (11)	−0.0039 (8)	0.0033 (9)	−0.0002 (9)
C14	0.0177 (11)	0.0132 (10)	0.0131 (10)	−0.0025 (8)	0.0019 (9)	0.0041 (9)
C15	0.0161 (11)	0.0143 (10)	0.0151 (11)	−0.0006 (8)	0.0021 (9)	0.0032 (9)
C16	0.0218 (12)	0.0164 (11)	0.0153 (11)	−0.0013 (9)	0.0025 (9)	−0.0013 (9)
C17	0.0240 (12)	0.0187 (12)	0.0209 (12)	0.0018 (10)	0.0070 (10)	−0.0015 (10)
C18	0.0142 (11)	0.0224 (12)	0.0283 (13)	0.0030 (9)	0.0036 (10)	0.0029 (10)
C19	0.0145 (10)	0.0216 (11)	0.0218 (13)	0.0010 (8)	0.0004 (9)	0.0027 (9)
C20	0.0148 (10)	0.0166 (10)	0.0173 (11)	−0.0028 (8)	0.0019 (8)	0.0033 (9)
C21	0.0144 (11)	0.0212 (12)	0.0157 (11)	−0.0030 (9)	0.0007 (9)	0.0011 (9)
C22	0.0171 (10)	0.0175 (11)	0.0135 (11)	−0.0024 (8)	0.0025 (8)	0.0051 (9)
C23	0.0174 (11)	0.0227 (12)	0.0199 (12)	−0.0047 (9)	−0.0023 (9)	0.0004 (10)
C24	0.0327 (14)	0.0209 (12)	0.0168 (12)	−0.0043 (10)	−0.0057 (10)	−0.0014 (10)
C25	0.0278 (12)	0.0192 (11)	0.0168 (11)	0.0025 (9)	−0.0008 (10)	−0.0006 (10)
C26	0.0186 (11)	0.0192 (11)	0.0181 (12)	0.0010 (9)	−0.0011 (9)	0.0017 (10)

### Geometric parameters (Å, °)

**Table:**

Mn1—N6	2.1764 (19)	C6—C7	1.361 (3)
Mn1—N7	2.1794 (18)	C6—H6	0.9500
Mn1—N1	2.2552 (19)	C7—H7	0.9500
Mn1—N5	2.2621 (19)	C8—H8A	0.9800
Mn1—N8	2.2684 (19)	C8—H8B	0.9800
Mn1—N3	2.280 (2)	C8—H8C	0.9800
O1—C14	1.255 (3)	C9—C10	1.381 (3)
O2—C21	1.252 (3)	C9—H9	0.9500
N1—C1	1.316 (3)	C10—C11	1.381 (4)
N1—C3	1.378 (3)	C10—H10	0.9500
N2—C1	1.335 (3)	C11—C12	1.387 (3)
N2—C2	1.371 (3)	C11—H11	0.9500
N2—C4	1.462 (3)	C12—C13	1.392 (3)
N3—C5	1.322 (3)	C12—H12	0.9500
N3—C7	1.378 (3)	C13—C14	1.513 (3)
N4—C5	1.340 (3)	C15—C16	1.408 (3)
N4—C6	1.369 (3)	C15—C20	1.427 (3)
N4—C8	1.461 (3)	C16—C17	1.389 (3)
N5—C9	1.343 (3)	C16—H16	0.9500
N5—C13	1.348 (3)	C17—C18	1.384 (3)
N6—C14	1.326 (3)	C17—H17	0.9500
N6—C15	1.413 (3)	C18—C19	1.387 (3)
N7—C21	1.327 (3)	C18—H18	0.9500
N7—C20	1.405 (3)	C19—C20	1.407 (3)
N8—C26	1.330 (3)	C19—H19	0.9500
N8—C22	1.349 (3)	C21—C22	1.526 (3)
C1—H1	0.9500	C22—C23	1.386 (3)
C2—C3	1.350 (3)	C23—C24	1.383 (3)
C2—H2	0.9500	C23—H23	0.9500
C3—H3	0.9500	C24—C25	1.382 (3)
C4—H4A	0.9800	C24—H24	0.9500
C4—H4B	0.9800	C25—C26	1.390 (4)
C4—H4C	0.9800	C25—H25	0.9500
C5—H5	0.9500	C26—H26	0.9500
			
N6—Mn1—N7	75.02 (7)	N3—C7—H7	125.0
N6—Mn1—N1	97.62 (7)	N4—C8—H8A	109.5
N7—Mn1—N1	99.09 (7)	N4—C8—H8B	109.5
N6—Mn1—N5	74.74 (7)	H8A—C8—H8B	109.5
N7—Mn1—N5	149.74 (7)	N4—C8—H8C	109.5
N1—Mn1—N5	85.93 (7)	H8A—C8—H8C	109.5
N6—Mn1—N8	149.72 (7)	H8B—C8—H8C	109.5
N7—Mn1—N8	74.73 (7)	N5—C9—C10	123.1 (2)
N1—Mn1—N8	88.21 (7)	N5—C9—H9	118.5
N5—Mn1—N8	135.47 (7)	C10—C9—H9	118.5
N6—Mn1—N3	103.48 (8)	C11—C10—C9	118.2 (2)
N7—Mn1—N3	104.27 (7)	C11—C10—H10	120.9
N1—Mn1—N3	151.79 (7)	C9—C10—H10	120.9
N5—Mn1—N3	81.85 (7)	C10—C11—C12	119.7 (2)
N8—Mn1—N3	82.97 (7)	C10—C11—H11	120.2
C1—N1—C3	104.67 (19)	C12—C11—H11	120.2
C1—N1—Mn1	130.57 (15)	C11—C12—C13	119.0 (2)
C3—N1—Mn1	124.52 (15)	C11—C12—H12	120.5
C1—N2—C2	106.58 (19)	C13—C12—H12	120.5
C1—N2—C4	127.1 (2)	N5—C13—C12	121.4 (2)
C2—N2—C4	126.3 (2)	N5—C13—C14	118.38 (19)
C5—N3—C7	104.76 (19)	C12—C13—C14	120.1 (2)
C5—N3—Mn1	123.51 (16)	O1—C14—N6	130.2 (2)
C7—N3—Mn1	130.40 (16)	O1—C14—C13	116.61 (19)
C5—N4—C6	107.1 (2)	N6—C14—C13	113.10 (19)
C5—N4—C8	125.9 (2)	C16—C15—N6	125.1 (2)
C6—N4—C8	127.0 (2)	C16—C15—C20	119.1 (2)
C9—N5—C13	118.7 (2)	N6—C15—C20	115.75 (19)
C9—N5—Mn1	127.17 (15)	C17—C16—C15	120.7 (2)
C13—N5—Mn1	112.77 (14)	C17—C16—H16	119.7
C14—N6—C15	123.67 (19)	C15—C16—H16	119.7
C14—N6—Mn1	118.88 (14)	C18—C17—C16	120.2 (2)
C15—N6—Mn1	116.61 (14)	C18—C17—H17	119.9
C21—N7—C20	123.51 (18)	C16—C17—H17	119.9
C21—N7—Mn1	119.67 (14)	C17—C18—C19	120.4 (2)
C20—N7—Mn1	116.81 (14)	C17—C18—H18	119.8
C26—N8—C22	118.98 (19)	C19—C18—H18	119.8
C26—N8—Mn1	126.43 (15)	C18—C19—C20	120.9 (2)
C22—N8—Mn1	112.66 (14)	C18—C19—H19	119.6
N1—C1—N2	112.5 (2)	C20—C19—H19	119.6
N1—C1—H1	123.7	N7—C20—C19	125.5 (2)
N2—C1—H1	123.7	N7—C20—C15	115.78 (18)
C3—C2—N2	106.4 (2)	C19—C20—C15	118.7 (2)
C3—C2—H2	126.8	O2—C21—N7	130.6 (2)
N2—C2—H2	126.8	O2—C21—C22	116.22 (19)
C2—C3—N1	109.8 (2)	N7—C21—C22	113.13 (19)
C2—C3—H3	125.1	N8—C22—C23	121.5 (2)
N1—C3—H3	125.1	N8—C22—C21	118.04 (19)
N2—C4—H4A	109.5	C23—C22—C21	120.44 (19)
N2—C4—H4B	109.5	C24—C23—C22	119.4 (2)
H4A—C4—H4B	109.5	C24—C23—H23	120.3
N2—C4—H4C	109.5	C22—C23—H23	120.3
H4A—C4—H4C	109.5	C25—C24—C23	119.0 (2)
H4B—C4—H4C	109.5	C25—C24—H24	120.5
N3—C5—N4	112.2 (2)	C23—C24—H24	120.5
N3—C5—H5	123.9	C24—C25—C26	118.6 (2)
N4—C5—H5	123.9	C24—C25—H25	120.7
C7—C6—N4	106.0 (2)	C26—C25—H25	120.7
C7—C6—H6	127.0	N8—C26—C25	122.6 (2)
N4—C6—H6	127.0	N8—C26—H26	118.7
C6—C7—N3	109.9 (2)	C25—C26—H26	118.7
C6—C7—H7	125.0		
			
N6—Mn1—N1—C1	−92.7 (2)	Mn1—N1—C3—C2	−174.18 (17)
N7—Mn1—N1—C1	−168.6 (2)	C7—N3—C5—N4	0.2 (3)
N5—Mn1—N1—C1	−18.7 (2)	Mn1—N3—C5—N4	−167.83 (14)
N8—Mn1—N1—C1	117.1 (2)	C6—N4—C5—N3	−0.3 (3)
N3—Mn1—N1—C1	45.6 (3)	C8—N4—C5—N3	−179.6 (2)
N6—Mn1—N1—C3	80.7 (2)	C5—N4—C6—C7	0.3 (3)
N7—Mn1—N1—C3	4.8 (2)	C8—N4—C6—C7	179.6 (2)
N5—Mn1—N1—C3	154.7 (2)	N4—C6—C7—N3	−0.1 (3)
N8—Mn1—N1—C3	−69.46 (19)	C5—N3—C7—C6	0.0 (3)
N3—Mn1—N1—C3	−140.97 (19)	Mn1—N3—C7—C6	166.84 (16)
N6—Mn1—N3—C5	34.70 (19)	C13—N5—C9—C10	−0.4 (3)
N7—Mn1—N3—C5	112.38 (18)	Mn1—N5—C9—C10	165.25 (17)
N1—Mn1—N3—C5	−102.6 (2)	N5—C9—C10—C11	1.5 (3)
N5—Mn1—N3—C5	−37.38 (18)	C9—C10—C11—C12	−1.0 (3)
N8—Mn1—N3—C5	−175.39 (18)	C10—C11—C12—C13	−0.5 (3)
N6—Mn1—N3—C7	−130.0 (2)	C9—N5—C13—C12	−1.2 (3)
N7—Mn1—N3—C7	−52.4 (2)	Mn1—N5—C13—C12	−168.82 (16)
N1—Mn1—N3—C7	92.6 (2)	C9—N5—C13—C14	175.61 (19)
N5—Mn1—N3—C7	157.9 (2)	Mn1—N5—C13—C14	8.0 (2)
N8—Mn1—N3—C7	19.9 (2)	C11—C12—C13—N5	1.6 (3)
N6—Mn1—N5—C9	−166.23 (19)	C11—C12—C13—C14	−175.1 (2)
N7—Mn1—N5—C9	−164.19 (16)	C15—N6—C14—O1	1.1 (4)
N1—Mn1—N5—C9	94.73 (18)	Mn1—N6—C14—O1	−168.07 (19)
N8—Mn1—N5—C9	11.5 (2)	C15—N6—C14—C13	−174.87 (18)
N3—Mn1—N5—C9	−59.78 (18)	Mn1—N6—C14—C13	16.0 (2)
N6—Mn1—N5—C13	0.09 (14)	N5—C13—C14—O1	167.57 (19)
N7—Mn1—N5—C13	2.1 (2)	C12—C13—C14—O1	−15.6 (3)
N1—Mn1—N5—C13	−98.95 (15)	N5—C13—C14—N6	−15.9 (3)
N8—Mn1—N5—C13	177.77 (13)	C12—C13—C14—N6	161.0 (2)
N3—Mn1—N5—C13	106.54 (15)	C14—N6—C15—C16	12.7 (3)
N7—Mn1—N6—C14	171.66 (18)	Mn1—N6—C15—C16	−177.88 (17)
N1—Mn1—N6—C14	74.24 (17)	C14—N6—C15—C20	−170.9 (2)
N5—Mn1—N6—C14	−9.40 (16)	Mn1—N6—C15—C20	−1.6 (2)
N8—Mn1—N6—C14	173.83 (15)	N6—C15—C16—C17	177.1 (2)
N3—Mn1—N6—C14	−86.90 (17)	C20—C15—C16—C17	0.9 (3)
N7—Mn1—N6—C15	1.75 (14)	C15—C16—C17—C18	−0.1 (3)
N1—Mn1—N6—C15	−95.67 (15)	C16—C17—C18—C19	−1.0 (4)
N5—Mn1—N6—C15	−179.31 (16)	C17—C18—C19—C20	1.2 (3)
N8—Mn1—N6—C15	3.9 (2)	C21—N7—C20—C19	−3.1 (3)
N3—Mn1—N6—C15	103.19 (15)	Mn1—N7—C20—C19	178.62 (17)
N6—Mn1—N7—C21	179.90 (17)	C21—N7—C20—C15	179.81 (19)
N1—Mn1—N7—C21	−84.58 (16)	Mn1—N7—C20—C15	1.5 (2)
N5—Mn1—N7—C21	177.87 (15)	C18—C19—C20—N7	−177.3 (2)
N8—Mn1—N7—C21	1.03 (15)	C18—C19—C20—C15	−0.3 (3)
N3—Mn1—N7—C21	79.48 (17)	C16—C15—C20—N7	176.57 (19)
N6—Mn1—N7—C20	−1.75 (14)	N6—C15—C20—N7	0.0 (3)
N1—Mn1—N7—C20	93.76 (15)	C16—C15—C20—C19	−0.7 (3)
N5—Mn1—N7—C20	−3.8 (2)	N6—C15—C20—C19	−177.28 (18)
N8—Mn1—N7—C20	179.38 (15)	C20—N7—C21—O2	−5.9 (4)
N3—Mn1—N7—C20	−102.17 (15)	Mn1—N7—C21—O2	172.31 (19)
N6—Mn1—N8—C26	168.94 (17)	C20—N7—C21—C22	173.58 (18)
N7—Mn1—N8—C26	171.1 (2)	Mn1—N7—C21—C22	−8.2 (2)
N1—Mn1—N8—C26	−88.97 (19)	C26—N8—C22—C23	−1.3 (3)
N5—Mn1—N8—C26	−6.6 (2)	Mn1—N8—C22—C23	163.93 (17)
N3—Mn1—N8—C26	64.18 (19)	C26—N8—C22—C21	−179.3 (2)
N6—Mn1—N8—C22	5.1 (2)	Mn1—N8—C22—C21	−14.1 (2)
N7—Mn1—N8—C22	7.25 (14)	O2—C21—C22—N8	−165.29 (19)
N1—Mn1—N8—C22	107.17 (15)	N7—C21—C22—N8	15.1 (3)
N5—Mn1—N8—C22	−170.47 (13)	O2—C21—C22—C23	16.7 (3)
N3—Mn1—N8—C22	−99.68 (15)	N7—C21—C22—C23	−162.9 (2)
C3—N1—C1—N2	−0.2 (3)	N8—C22—C23—C24	−0.4 (3)
Mn1—N1—C1—N2	174.15 (16)	C21—C22—C23—C24	177.6 (2)
C2—N2—C1—N1	−0.3 (3)	C22—C23—C24—C25	1.9 (3)
C4—N2—C1—N1	178.6 (2)	C23—C24—C25—C26	−1.8 (4)
C1—N2—C2—C3	0.6 (3)	C22—N8—C26—C25	1.4 (3)
C4—N2—C2—C3	−178.2 (2)	Mn1—N8—C26—C25	−161.57 (18)
N2—C2—C3—N1	−0.8 (3)	C24—C25—C26—N8	0.2 (4)
C1—N1—C3—C2	0.6 (3)		
